# Genome-wide transcriptomic analysis of the response to nitrogen limitation in *Streptomyces coelicolor *A3(2)

**DOI:** 10.1186/1756-0500-4-78

**Published:** 2011-03-23

**Authors:** Richard A Lewis, Sanjay K Shahi, Emma Laing, Giselda Bucca, Georgios Efthimiou, Michael Bushell, Colin P Smith

**Affiliations:** 1 Faculty of Heath & Medical Sciences, University of Surrey, Stag Hill, Guildford, Surrey, GU2 7XH, UK

## Abstract

**Background:**

The present study represents a genome-wide transcriptomic analysis of the response of the model streptomycete *Streptomyces coelicolor *A3(2) M145 to fermentor culture in Modified Evans Media limited, respectively, for nitrogen, phosphate and carbon undertaken as part of the ActinoGEN consortium to provide a publicly available reference microarray dataset.

**Findings:**

A microarray dataset using samples from two replicate cultures for each nutrient limitation was generated. In this report our analysis has focused on the genes which are significantly differentially expressed, as determined by Rank Products Analysis, between samples from matched time points correlated by growth phase for the three pairs of differently limited culture datasets. With a few exceptions, genes are only significantly differentially expressed between the N6/N7 time points and their corresponding time points in the C and P-limited cultures, with the vast majority of the differentially expressed genes being more highly expressed in the N-limited cultures. Our analysis of these genes indicated expression of several members of the GlnR regulon are induced upon nitrogen limitation, as assayed for by [NH_4_^+^] measurements, and we are able to identify several additional genes not present in the GlnR regulon whose expression is induced in response to nitrogen limitation. We also note SCO3327 which encodes a small protein (32 amino acid residues) unusually rich in the basic amino acids lysine (31.25%) and arginine (25%) is significantly differentially expressed in the nitrogen limited cultures. Additionally, we investigate the expression of known members of the GlnR regulon and the relationship between gene organization and expression for the SCO2486-SCO2487 and SCO5583-SCO5585 operons.

**Conclusions:**

We provide a list of genes whose expression is differentially expressed in low nitrogen culture conditions, including a putative nitrogen storage protein encoded by SCO3327. Our list includes several genes whose expression patterns are similar to up-regulated members of the GlnR regulon and are induced in response to nitrogen limitation. These genes represent likely targets for future studies into the nitrogen starvation response in *Streptomyces coelicolor*.

## Introduction

It has long been known that the carbon (C), nitrogen (N) and phosphate (P) ratios of *Streptomyces *media require optimization for secondary metabolite biosynthesis [1,2]. As the soil, the natural habitat of the model streptomycete *Streptomyces coelicolor*, is carbon rich, investigations have focused upon determining the affect on metabolism and gene regulation of limiting the supply of the other two key nutrients, phosphate and nitrogen and *Streptomyces *nitrogen metabolism has been previously reviewed [3,4]. Of the five genes in *S. coelicolor *which are predicated to encode glutamine synthetase only two (*glnA *&*glnII*) [5] are believed to be functional whilst the others (*glnA2*, *glnA3 *&*glnA4*) are redundant and lack conserved residues involved in catalysis [6]. The housekeeping GSI type enzyme GlnA is expressed at a constant level throughout development [7] and the GSII type enzyme, GlnII, is thought to play a specific role in development as it is active only during differentiation of mycelium on solid growth media [8,9]. Following an increase in the NH_4_^+ ^supply GlnA activity is reduced due to it being adenylylated by the adenylyltransferase enzyme, GlnE, and conversely its activity may be restored by de-adenylylation following a decrease in the NH_4_^+ ^supply [7,10]. In *E. coli *GlnE activity is regulated by GlnB, GlnD and GlnK and although homologues of GlnD and GlnK have been identified in *S. coelicolor *their deletion does not affect the regulation of GlnA activity [11]. Under nitrogen limiting conditions GlnD post-translationally modifies the PII protein, GlnK, by adenylylating a conserved tyrosine residue. Furthermore, although this modification is reversible, a secondary modification, the removal of the N-terminal three amino acids of GlnK is not reversible following a nitrogen starvation shock [11]. Therefore, although it is clear that differences in nitrogen availability result in changes to GlnD/K activity, the physiological significance of this phenomenon is unknown. Similarly, the physiological significance of a ncRNA which interferes with the translation of *glnA *mRNA is also unclear [12]. In addition to post-translational modifications genes involved in nitrogen metabolism also seem to be regulated at the level of transcription. The master regulator GlnR [13] has been shown to interact with a consensus sequence present in the upstream regions of *glnA *and the *amtB-glnK-glnD *operon and to up-regulate GlnA activity [9,14]. A second potential regulator, GlnRII, was also identified which bound the promoter regions of the *amtB *operon, *glnA *and *glnII*, although its functional significance is unclear [9]. More recent studies have extended the GlnR regulon to encompass further genes involved in nitrogen metabolism *i.e*. the glutamine dehydrogenase gene (*gdh*; SCO4683), the nitrate reductase gene (*nirB*; SCO2486) and urease gamma subunit (*ureA*; SCO1236), in addition to other genes whose involvement in the nitrogen starvation response is unclear [15]. An assimilatory nitrate reductase encoding gene, *nasA *(SCO2473), whose promoter possesses a variant of the GlnR binding sequence was also identified [16]. It is likely that the nitrogen and phosphate transcriptional regulatory networks are linked as it has been shown that PhoP represses transcription from the *glnR*, *glnA*, *glnII *and *amtB *promoters, which possess PhoP binding sites [17]. This interaction was first suggested by microarray data [18] and to extend these studies and enhance our understanding of the interactions of different nutrient specific regulatory networks the present study was conceived under the aegis of the European ActinoGEN consortium.

The work presented here was designed to use microarray technology to characterize the differences in transcription which occur in fermentor cultures of the wild-type *S. coelicolor *A3(2) strain M145 grown in defined, minimal liquid media limited for nitrogen, carbon and phosphate, respectively and provide a publicly available reference dataset. To our knowledge no microarray data relating to *Streptomyces coelicolor *gene expression under carbon-limited culture conditions have been published. Additionally, the present study represents an advance over earlier microarray studies of gene expression in phosphate limited culture, both in terms of sampling frequency and standardization of culture conditions [18], and also represents an advance over previous studies of gene expression in nitrogen limited culture, both in terms of number of replicates, sampling frequency and gene coverage [15].

### Hypothesis

Growth of *Streptomyces coelicolor *A3(2) M145 in fermentor cultures limited for nitrogen results in a set of genes being differentially expressed when compared with similar cultures limited for phosphate and carbon respectively.

## Materials and methods

### Strain

In this study the *Streptomyces coelicolor *A3(2) strain M145 was used which is a prototrophic derivative of strain A3(2) lacking its two plasmids, SCP1 and SCP2. This is the standard strain used by the ActinoGEN consortium and the strain from which the *Streptomyces coelicolor *genome sequence was obtained [19].

### Media preparation

Cultures were performed using the following media:- Mannitol-Soya (MS) agar [20]. GGI:- Glucose 15 g, Glycerol, 15 g, soya peptone, 15 g, NaCl_2 _3 g, CaCO_3 _1 g, dH_2_O to 1 L followed by adjustment of the pH to 6.8 and sterilization by autoclaving. GYB:- Glucose 33 g, yeast extract 15 g, dH_2_O to 1 L followed by adjustment of the pH to 6.8 and sterilization by filter sterilization. P, N and C- limited Modified Evans Media were prepared as set out in Additional File 1. Glucose solution concentrates (N, P-limited: 700 mM, C-limited: 250 mM) were autoclaved separately from the other mixed media components and added aseptically. Five ml/L of Trace Elements (ZnO 50 mM, FeCl_3 _20 mM, MnCl_2 _10 mM, CuCl_2 _10 mM, CoCl_2 _20 mM, H_3_BO_3 _10 mM, Na_2_MoO_4 _0.02 mM, HCl 80 ml, dH_2_0 up to 1 L) was also added aseptically to the other media components following autoclaving.

### Inoculum preparation

A dense spore suspension was streaked out to produce a confluent lawn on MS agar. Following sporulation a plug was cut with the wide end of a 1 ml pipette tip and used to inoculate 50 ml of GGI medium in a 250 ml flask which was incubated for 48 hr at 30°C with agitation (200 rpm). Four ml of this culture was subsequently used to inoculate 50 ml of GYB medium which was incubated for 24 h at 30°C with agitation (200 rpm). The GYB culture was subsequently used to inoculate (10% final volume) Modified Evans Medium (see Additional File 1) which was incubated for 24 h at 30°C with agitation (200 rpm). This culture was used as the fermentor inoculum (4% v/v). The three step procedure ensured minimal carry-over of rich nutrients from the inoculum production flasks into the fermentor.

### Bioreactor culture and sampling

The inoculum culture (4%v/v) was used to inoculate a 5 L fermentor vessel (Adaptive Biosystems, Luton, UK) containing 3 L of Modified Evans Medium. Temperature was maintained at 30°C, pH at 6.8, agitation (800 rpm) was achieved using a large marine impeller and the aeration rate was 2 L/min. All cultures were performed in duplicate *i.e*. the "Inoculum Preparation" and "Bioreactor Culture and Sampling" protocols were repeated for each type of Modified Evans Medium to generate two independent sets of replicate samples per medium type. Samples were taken for biomass determination and RNA isolation at intervals, which were more frequent during the exponential and first transition phases. Three × five ml samples were filtered through 0.45 μm predried, preweighed nitrocellulose membranes (Millipore, Watford, UK) and the culture filtrate was retained for actinorhodin assay [21] and nutrient analyses (PO_4_^-^, NH_4_^+ ^and Glucose) using a Merck RQFlex10™ reflectometer. The nitrocellulose membranes were rinsed three times with ultrapure water, microwaved twice at 650 W for five min and allowed to cool in a dessicator before the weight was determined. For RNA isolation culture samples were added to two volumes of RNAprotect (Qiagen) in a Falcon tube and vortex-mixed. The mixture was incubated for 10 min at room temperature before centrifugation at 3500 rpm for 10 min. The supernatant was decanted and any excess liquid removed by inverting the tube before storage of the cell pellet at -80°C. For a limited number of time points cell pellets derived from 5 ml of culture were harvested for undecylprodiginine assay [22].

### Nucleic acid isolation and microarray techniques

Protocols for RNA isolation using the "Total RNA extraction by Tissue Lyzer" are provided at our microarray resource website [23]. The quality of the RNA obtained was assessed by running a Bioanalyzer Prokaryote Total RNA Nano chip (Agilent) and only RNA having a RNA Integrity Number of 6.5 or above was used in subsequent work. Protocols for *S. coelicolor *M145 genomic DNA isolation and labeling (Cy5), together with the labeling of cDNA (Cy3) are as described at our microarray resource website [23] and hybridizations were set up using our own in-house printed *Streptomyces coelicolor *oligonucleotide based spotted microarrays [23], as described at the microarray resource website [23].

### Microarray data processing and analysis

The microarrays were scanned using an Agilent scanner (5 μm resolution).

**Figure 1:**
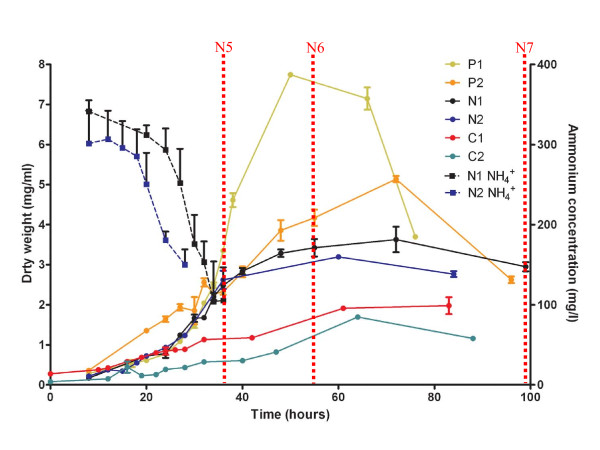
**Growth curves for duplicate fermentor cultures of *S. coelicolor *A2(3) M145 grown in N, P and C-limited Modified Evans Medium**. Three dry weights per sample per time point were averaged to provide each datum. To align the exponential phases of the cultures with the N1 culture the time points for the N2, C1 and C2 cultures have been shifted by -12, -8 and -8 h respectively. The [NH_4_^+^] assay data (where available) for the N-limited cultures are also shown. The N5-7 time points, aligned to the N1 growth curve, are indicated by dashed red bars.

Images were imported into the software BlueFuse (Version 3.1; BlueGnome Ltd, Cambridge) for feature extraction and spot quantification as detailed in [24]. BlueFuse output files were imported into the statistical computing environment R (Version 2.5.1) [25,26] for data normalization using the Limma package [27,28]. Within-array global median normalization of log_2 _cDNA/gDNA ratios was applied to each array in the analysis, ignoring control and flagged spots. Subsequent log_2 _ratios were scaled to have the same median-absolute-deviation (MAD) across all arrays [27,28]. Flagging was applied to the normalized data such that probes with a PON CH2 (probability of a good signal in the genomic DNA channel of the array, as designated by BlueFuse) value less than 0.5 were assigned 'NA'. Genes that did not have at least one good probe for each condition/replicate were filtered out and not included in any downstream analysis. Due to the high stringency of this filtering only 5,060 genes, which gave a signal in all of the 38 samples used in the study, were analyzed using Rank products analysis [29]. The R package RankProd [30] was used to perform Rank products analysis with default parameters for each pairwise condition/time-point comparison in the dataset. Following this, lists of significantly (Rank Products pfp value of ≤0.15) differentially expressed genes were identified for each pair of time points. In this report our analysis has focused on the genes which are significantly differentially expressed between samples from matched time points (T1-T7) for the three pairs of datasets *i.e*. N vs C, N vs P, and C vs P as set out in Additional File 2. Alignment of the growth curves (Figure 1) ensured the matched time points from differently limited cultures were correlated by growth phase. Although seven time points from the C and N-limited cultures could be matched to one another, due to the lower sampling frequency for the P-limited cultures only five time points could be matched with corresponding pairs of time points from the N and C-limited cultures. This approach was adopted so as to nullify the effect of interference from growth phase-dependent gene expression on the results and ensure that the lists of significantly differentially expressed genes generated are directly attributable to differences in nutrient limitation. The microarray data has been deposited with ArrayExpress (Accession number: E-MAXD-59).

## Results and Discussion

The growth curves of the cultures grown in N, P and C-limited media are strikingly different, (Figure 1) as are the amounts of actinorhodin and undecylprodiginine produced (see Additional Files 3, 4 &5). The C-limited cultures produced less biomass and pigmented antibiotics than the N and P-limited cultures whereas the P-limited cultures gave the highest biomass readings and production of actinorhodin and undecylprodiginine. It is clear from the metabolite measurements that the NH_4_^+ ^, PO_4_^-^, and glucose levels in the N, P, C-limited cultures decrease during the culture period to low, and presumably, limiting levels (see Additional File 6).

Examination of the gene lists obtained from Rank Products analysis indicate that no genes are significantly differentially expressed between the C and P-limited cultures. Moreover, with a few exceptions, genes are only significantly differentially expressed between the N6 and N7 time points and their corresponding time points in the C and P-limited cultures, with the vast majority of the genes being more highly expressed in the N limited cultures. Most of the significantly differentially expressed genes were identified as such at both the N6 and the N7 time points in both the N vs C and the N vs P comparisons. It appears, *prima facie*, that either our cultures were more successfully limited with respect to nitrogen than they were with respect to carbon or phosphate, or that genes which are differentially expressed in P and C-limited media are not represented in our dataset, or that nitrogen limitation has a greater effect on differential gene expression than either carbon or phosphate limitation. Our analysis of the present dataset, focusing on the genes which are significantly differentially expressed between the N6 and N7 time points and their corresponding time points in the C and P-limited cultures (see Additional File 7), was conducted according to the last premise.

The results indicated that the majority of genes may be categorized into three main gene Expression Categories (I, II or III) as follows. Firstly, (I) genes which have fairly constant, elevated expression in the N-limited cultures relative to the P-limited cultures, and whose expression is also low, or decreases with time across the C-limited cultures. Secondly (II) genes whose expression increases relatively uniformly across the N-limited culture time points to an elevated level relative to the C, and P- limited cultures. Thirdly, (III) and most interestingly, genes whose expression is elevated in the N6 and/or N7 time points relative to both the early N-limited culture time points and the C and P-limited cultures.

**Figure 2:**
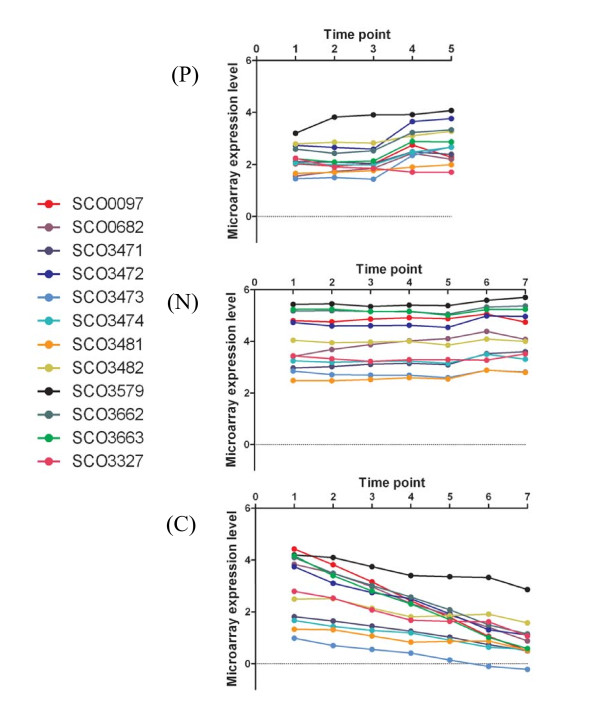
**Microarray expression data for Expression Category (I) genes averaged for matched time points between duplicate cultures *S. coelicolor *A2(3) M145 grown in (P) P-limited Modified Evans Medium; (N) N-limited Modified Evans Medium, (C) C-limited Modified Evans Medium**.

**Figure 3:**
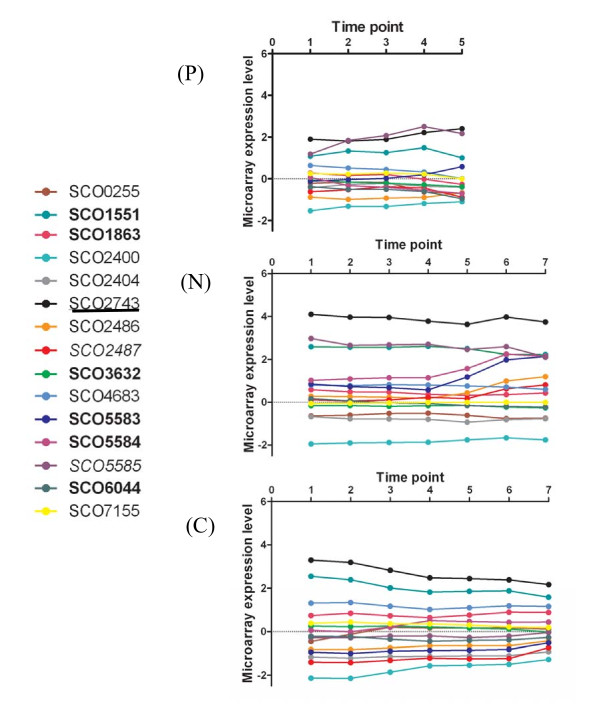
**Microarray expression data for genes of the GlnR regulon averaged for matched time points between duplicate cultures *S. coelicolor *A2(3) M145 grown in (P) P-limited Modified Evans Medium; (N) N-limited Modified Evans Medium, (C) C-limited Modified Evans Medium**. Genes identified as GlnR regulated in [9] are shown in **bold **text, genes identified in [15] are shown in plain text; genes identified in [16] are shown in underlined text and genes of interest identified during the present study, but not previously included in the GlnR regulon are shown in *italics*.

Regarding the genes of Expression Category (I), clearly, the genes have been identified through their high expression under nitrogen limiting conditions relative to the other limited cultures (Figure 2). However their expression patterns do not resemble those of the GlnR regulon up-regulated genes (see below) and their annotations do not suggest involvement in nitrogen metabolism. For example, the regulatory WblA-encoding gene, SCO3579, which is a pleiotropic down-regulator of antibiotic biosynthesis [31,32] is present in this category. However, we note that SCO3327 (Figure 2) encodes a small (32 amino acid) protein which comprises an unusually high proportion of basic amino acid residues (>50%) with long chain R groups containing nitrogen *i.e*. arginine (25%) and lysine (31.25%). Two proteins rich in arginine have previously been identified whose genes have promoters which comprise putative GlnR binding sites *i.e*. SCO1863 is a 406 amino acid protein (see Figure 3) comprising 83 arginine residues (20.4%) [9], and SCO2195 is a 71 amino acid protein comprising 13 arginines (18.3%) [15]. Although all three proteins lack direct sequence similarity with one another they are similar in terms of having a high percentage of arginine residues, which is considerably higher than the standard *S. coelicolor *protein arginine composition of 8% [20]. The role/s of these proteins in nitrogen limited metabolism are unclear, although it has been suggested that SCO2195 is involved in RNA binding [15] and that SCO1863 may be a nitrogen storage protein [3] reflecting the fact that arginine is the most nitrogen rich amino acid comprising three atoms of nitrogen per residue.

It is clear that many of the genes comprising Expression Category (I) are involved in carbohydrate metabolism. For example, they include an operon comprising *dagA *(SCO3471) which encodes an extracellular agarase, a putative aldolase (SCO3473), a sugar kinase (SCO3474) together with the co-expressed SCO3472 (a transposase remnant). A glycosyltransferase homologue SCO3481 and a sugar permease (SCO3482) are also presumably expressed as an operon. The down regulation of these carbohydrate metabolism genes in the carbon limited culture may be a response to lack of substrate, however, we speculate that the differential expression of these genes under different nutrient limitations is due to a complex network of regulatory interactions which we are only now beginning to explore. For example, it is known that there are links between nitrogen and carbon metabolism, for example, mediated by *dasR *[33] and the links between GlnR and PhoP regulatory networks are mentioned above [17,18].

**Figure 4:**
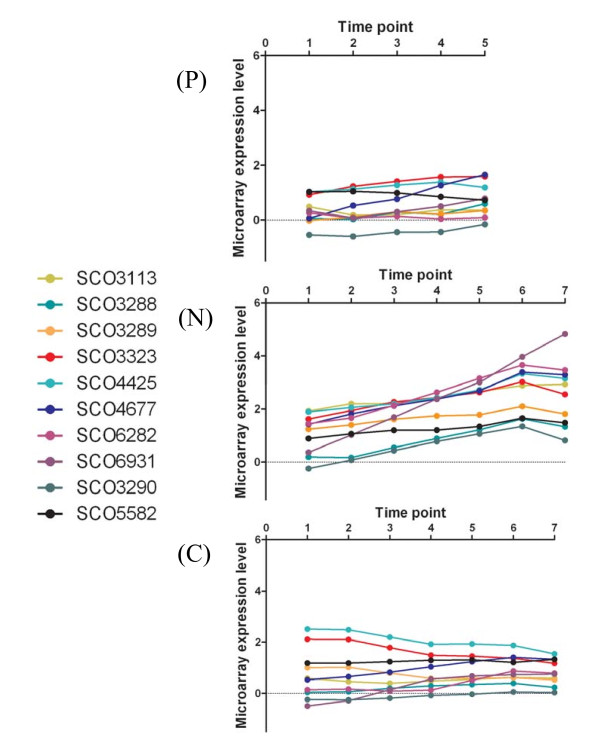
**Microarray expression data for Expression Category (II) genes averaged for matched time points between duplicate cultures *S. coelicolor *A2(3) M145 grown in (P) P-limited Modified Evans Medium; (N) N-limited Modified Evans Medium, (C) C-limited Modified Evans Medium**.

The second Expression Category of genes (II) comprises several regulatory genes, for example, the anti-sigma factor, SCO4677 which is involved in differentiation [34] and the sigma factor-like SCO3323 (see Figure 4). Genes involved in antibiotic biosynthesis/regulation are also represented and include the actinorhodin transporter SCO5083 (data not shown) and the transcriptional regulator *afsR2 *(SCO4425).

Several of the significantly differentially expressed genes of the third Expression Category (III) are known to be members of the GlnR regulon and are involved in nitrogen metabolism (see Figure 3). These include the nitrite reductase gene, *nirB *(SCO2486) together with the ammonium transporter protein *amtB *(SCO5583) and the gene encoding the PII protein *glnK *(SCO5584). Examination of their expression profiles indicates that their expression increases between time points N5 and N6 and is not a transient phenomenon as their expression remains elevated into N7. This phenomenon may be explained by consideration of the NH_4_^+ ^concentration data (Figure 1) which indicates that the ammonium ion concentration falls steeply in the nitrogen limited cultures during exponential phase to concentrations below the detection limits of the assay. Presumably this decrease in available nitrogen leads to the culture becoming nitrogen limited during the T5-T6 interval, so triggering the up-regulation of genes involved in nitrogen uptake and metabolism.

Although they are not significantly differentially expressed other genes of the GlnR regulon which are present in our dataset include the NADP-specific glutamate dehydrogenase gene *gdh *(SCO4683), SCO0255, SCO2400, SCO2404, SCO1863, SCO7155 and the assimilatory nitrate reductase gene *nasR *(SCO2473) [16]. Their expression profiles together with the other members of the GlnR regulon represented in the dataset are presented in Figure 3 (genes identified as GlnR-regulated in [9] are shown in **bold **text, genes identified in [15] are shown in plain text; genes identified in [16] are shown in underlined text). Given the different culture conditions used and gene expression determination methodologies employed it is difficult to compare our expression data with previous results relating to the transcription of these genes [9,15,16]. However, we note that our results regarding up-regulation of SCO2486 and SCO5583/5584 in nitrogen limiting conditions are consistent with previous data [9,15]. Additionally, our data indicating the non-alteration of expression of SCO7155 under nitrogen limitation conditions is consistent with previous results [15] and our results which show SCO0255, SCO2400 and SCO2404 are being expressed at a constant, low level are also consistent with the previously reported repression of these genes under nitrogen limiting conditions [15].

**Figure 5:**
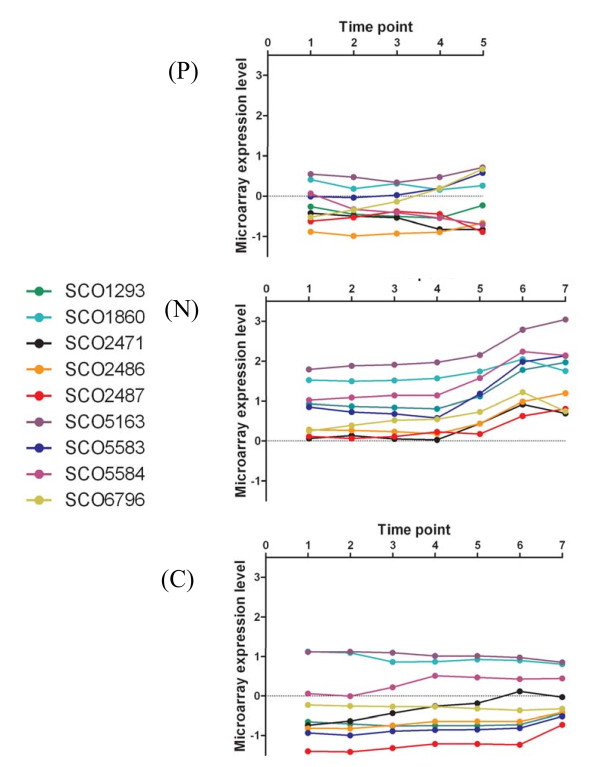
**Microarray expression data for Expression Category (III) genes averaged for matched time points between duplicate cultures *S. coelicolor *A2(3) M145 grown in (P) P-limited Modified Evans Medium; (N) N-limited Modified Evans Medium, (C) C- limited Modified Evans Medium**.

We examined the expression profiles of the other significantly differentially expressed genes to identify other genes whose expression patterns are similar to those of SCO2486, SCO5583 and SCO5584 and whose expression may be induced as a result of nitrogen limitation. The expression profiles of these genes are shown in Figure 5. Furthermore, we have also identified genes which are significantly differentially expressed between the N1 time point and successive N-limited culture time points. The majority of these were already represented in the gene lists of significantly differentially expressed genes identified as a result of comparing the N limited cultures with those of the C and P limited cultures (see Additional File 7). However, an additional five genes were identified (see Additional File 7) whose expression patterns are consistent with them being members of Expression Category II. These include SCO3290 (Figure 4) whose expression profile correlates with those of SCO3288 & SCO3289 (Figure 4) which are present in our list of differentially regulated genes. We cannot however exclude the possibility that the expression of these five genes is growth phase dependent and is induced on entry into stationary phase. For example, although SCO5582 expression is up-regulated (Figure 4) it seems to encode a regulator with sequence similarity to a sporulation associated protein.

We have analyzed the upstream regions (up to 750 bp) of all of the significantly differentially expressed genes identified in the present study for potential transcription factor/repressor binding motifs using the sequence motif algorithims MEME [35] and GLAM2, [36] in addition to manual inspection of the sequences. Barring the GlnR binding sites known to exist upstream of members of the GlnR regulon we have been unable to identify further potential protein binding target sequences, excepting two potential "a-site" motifs (GTAAC & GTAAT) separated by 17 nt upstream of SCO5163 (-141 to -168) which may represent a *nasA *type GlnR-binding element [16].

We have also used the STRING 8.2 database of protein-protein interactions [37] to search for known interactions between the genes. We note that SCO1861 is linked to SCO5583 and SCO5584 and that SCO1860 is present in our list of significantly differentially expressed genes whilst SCO1863 (the first gene in the SCO1863-1860 operon) has been identified as possessing a putative upstream GlnR binding site [9]. Barring other obvious associations *i.e*. consecutive genes present in operons or well known groupings *i.e*. the GlnR regulon we have been unable to identify further interactions between the significantly differentially expressed genes products.

The gene annotations of the majority of the significantly differentially expressed genes are insufficiently detailed to allow their assignment to specific roles in nitrogen limited metabolism. However, we note that SCO1293 whose expression appears to be induced following nitrogen limitation (Figure 5N) encodes a product with sequence similarity to N-acetylglutamate synthetases. Interestingly also we find that the nitrate reductase large subunit gene, *nirB*, (SCO2487) is significantly differentially expressed and that its expression profile closely matches that of the upstream nitrite reductase gene *nirB *(Figure 5N) which is known to be regulated by GlnR. This result is consistent with the arrangement of the genes in the chromosome as the SCO2487 coding sequence overlaps SCO2486, and the two genes are presumably co-expressed in an operon. We therefore propose the inclusion of SCO2487 in the GlnR regulon.

Similarly, we also note that the expression profiles of SCO5583 and SCO5584 (Figure 5N) closely match one another which are consistent with previously reported (RT)-PCR results [9]. Our data are also consistent with genetic organization *i.e*. the sequences of SCO5583 and SCO5584 overlap and are co-transcribed in a single mRNA [9]. Although the gene immediately downstream, *i.e. glnD *(SCO5585) which encodes an adenylyltransferase responsible for regulating GlnK activity *via *post translational modification is present in our list of significantly differentially expressed genes it appears from our data that its expression profile differs from those of SCO5583 and SCO5584. Interestingly, although it has been shown that SCO5584 and SCO5585 are transcriptionally linked the presence of an additional SCO5585 promoter was not ruled out [9] and our results tend to suggest that the presence of an additional promoter is indeed likely explaining the differential regulation of SCO5585 with respect to SCO5583 & SCO5584 (Figure 3N).

## Conclusions

The present study represents an investigation designed to identify genes of interest involved in *Streptomyces *nitrogen metabolism which could not be identified simply by sequence similarity searches. In this aim we were successful and we identify a number of genes as targets for future investigation. Of particular interest are the putative nitrogen storage protein encoded by SCO3327, and the up-regulated genes SCO1293 and SCO5163 which may play roles in the nitrogen starvation response. We expect that the present study will serve to inform and stimulate future research into the genes identified herein.

## Competing interests

The authors declare that they have no competing interests.

## Authors' contributions

RAL did the microarray experiments, metabolite assays, microarray data analysis and wrote the manuscript. EL processed the microarray data and contributed to the manuscript. SKS did the fermentor cultures. GB assisted with the microarray experiments and microarray data analysis. GE did the pigmented antibiotic assays. MB assisted with the fermentor experiments. CPS designed the study and directed the work. All of the authors have read and approved the final manuscript.

## Supplementary Material

Additional File 1 **Table providing details of the Modified Evans Media (P, C and N-limited) used in the present study**. Click here for file

Additional File 2 **Table providing details of matched pairs of samples from duplicate cultures *S. coelicolor *A2(3) M145 grown in differently limited (N, P, C) Modified Evans Medium and correlated between the differently limited cultures according to time point *i.e*. growth phase**. Click here for file

Additional File 3 **Results of actinorhodin assays of samples from *S. coelicolor *A2(3) M145 cultures grown in differently limited (N, P, C) Modified Evans Medium**. Data are averaged results from two technical replicates. Click here for file

Additional File 4 **Results of undecylprodiginine assays of samples from *S. coelicolor *A2(3) M145 cultures grown in differently limited (N, P, C) Modified Evans Medium**. Data are averaged results from two technical replicates. Click here for file

Additional File 5 **Graph of results of actinorhodin and undecylprodiginine assays of samples from *S. coelicolor *A2(3) M145 cultures grown in differently limited (N, P, C) Modified Evans Medium**. Click here for file

Additional File 6 **Results of phosphate, ammonium and glucose assays of samples from *S. coelicolor *A2(3) M145 cultures grown in differently limited (N, P, C) Modified Evans Medium**. "ND" indicates the concentration was below the detection threshold of the assay system. Data are averaged results from at least two technical replicates with the exception of the P2 phosphate assay which represents the results of a single replicate. Click here for file

Additional File 7 **List of genes significantly differentially expressed at the N6/N7 time points relative to comparison time points**. Details of their functional annotations and Expression Category (I, II or III) as defined in the "Results & Discussion" are provided. Those genes identified as a result of comparing the C-limited time points with the N-limited time points are marked *, those identified as a result of comparing the P-limited time points with the N-limited time points are marked # and those identified as a result of comparing the N1 nitrogen time point with subsequent N-limited time points are marked +. Unmarked genes were identified as a result of comparisons between the N- limited time points and both the C and P-limited time points. Click here for file
