# Supplementary material for: Genome-wide transcriptomic analysis of the response to nitrogen limitation in Streptomyces coelicolor A3(2)
Source: BMC Res Notes. 2011 Mar 23;4:78. doi: 10.1186/1756-0500-4-78 (PMC3073908; doi:10.1186/1756-0500-4-78)
Supplement: Additional File 1 — Table providing details of the Modified Evans Media (P, C and N-limited) used in the present study. [file 1756-0500-4-78-S1.DOC]

**Additional File 1**

|  | **Composition** | | | |
| --- | --- | --- | --- | --- |
| **Chemicals** | **P-limited**  **mM** | **C-limited**  **mM** | **N- limited**  **mM** | **Modified Evans Medium**  **mM** |
| NaH_2_PO_4._H_2_O  FW : 138  (Monohydrate, Sigma) | 4 | 10 | 10 | 10 |
| KCl  FW : 74.56  (Anhydrous Fisher) | 10 | 10 | 10 | 10 |
| MgCl_2_.6 H_2_O  FW : 203.31  (Hexahydrate Fisher) | 1.25 | 1.25 | 1.25 | 1.25 |
| (NH_4_)_2_SO_4_  FW 132.14  (Anhydrous Fisher) | 50 | 50 | 10 | 50 |
| Na_2_SO­_4_  FW: 142.04  (Anhydrous Fisher) | 2 | 2 | 2 | 2 |
| Citric Acid  FW : 192.1  Anhydrous Sigma) | 2 | 2 | 2 | 2 |
| CaCl_2_  FW : 111  (Anhydrous Sigma) | 0.25 | 0.25 | 0.25 | 0.25 |
| Glucose  FW: 180 | 140 | 50 | 140 | 140 |
| Trace Elements^2^ | 5ml/l | 5ml/l | 5ml/l | 5ml/l |
