# Supplementary material for: Genome-wide transcriptomic analysis of the response to nitrogen limitation in Streptomyces coelicolor A3(2)
Source: BMC Res Notes. 2011 Mar 23;4:78. doi: 10.1186/1756-0500-4-78 (PMC3073908; doi:10.1186/1756-0500-4-78)
Supplement: Additional File 2 — Table providing details of matched pairs of samples from duplicate cultures S. coelicolor A2(3) M145 grown in differently limited (N, P, C) Modified Evans Medium and correlated between the differently limited cultures according to time point i.e. growth phase. [file 1756-0500-4-78-S2.DOC]

**Additional File 2**

| P- Limited Culture  Time points | N- Limited Culture  Time points | C- Limited Culture  Time points |
| --- | --- | --- |
| - | T1 (N1:8; N2:20) | T1 (C1:20; C2:20) |
| - | T2 (N1:20; N2;30) | T2 (C1:30; C2:30) |
| T1 (P1:24; P24) | T3 (N1:24; N2:36) | T3 (C1:32; C2:32) |
| T2 (P1:30; P2:30) | T4 (N1:27; N2:40) | T4 (C1:34; C2:36) |
| T3 (P1:26; P2:36) | T5 (N1:36; N2:48) | T5 (C1:40; C2:40) |
| T4 (P1:47; P2:48) | T6 (N1:55; N2:72) | T6 (C1:50; C2:48) |
| T5 (P1:54; P2:55) | T7 (N1:96; N2:120) | T7 (C1:69; C2:72) |
