# Supplementary material for: Genome-wide transcriptomic analysis of the response to nitrogen limitation in Streptomyces coelicolor A3(2)
Source: BMC Res Notes. 2011 Mar 23;4:78. doi: 10.1186/1756-0500-4-78 (PMC3073908; doi:10.1186/1756-0500-4-78)

Additional File 5

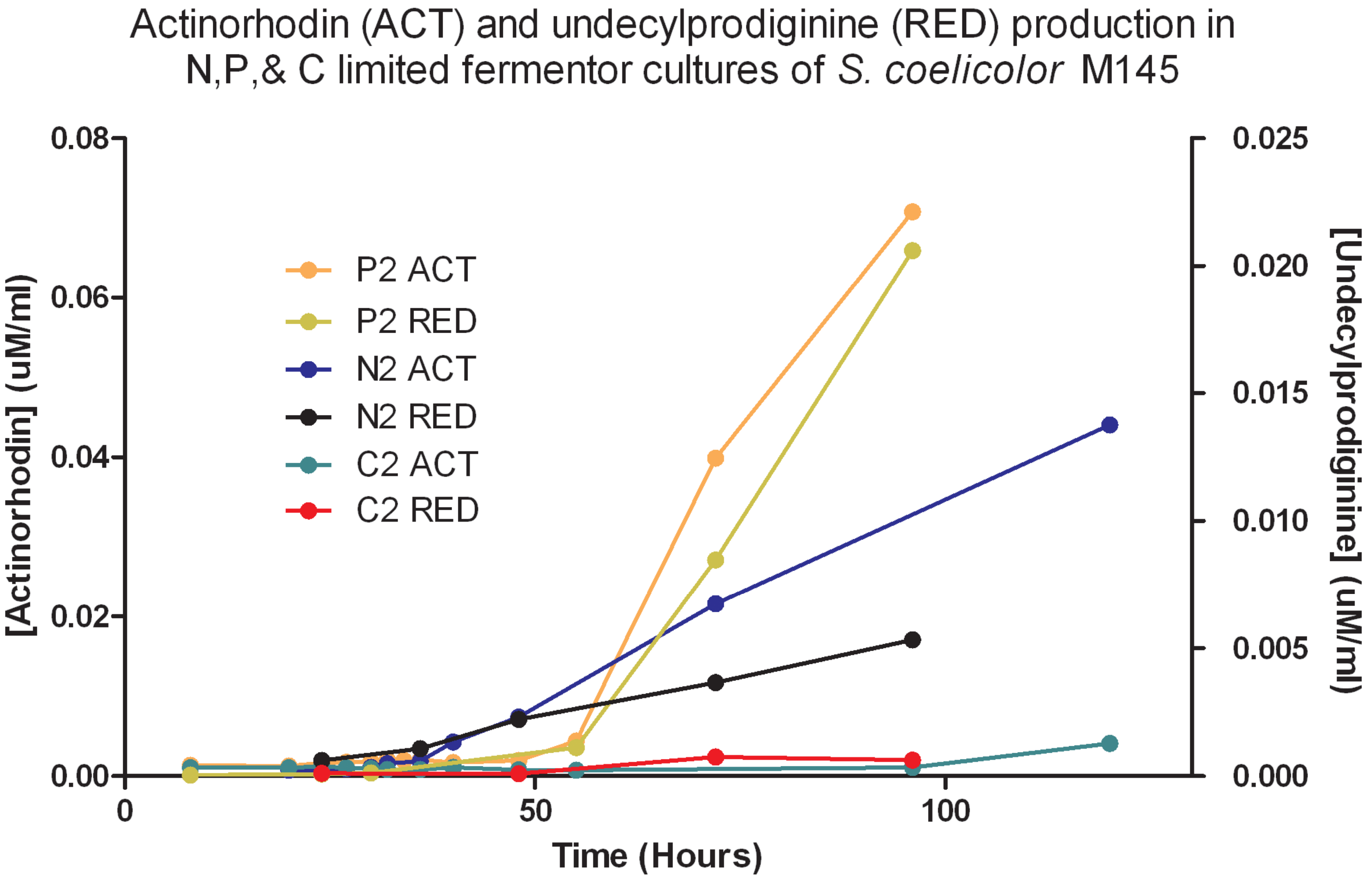

Supplement: Additional File 5 — Graph of results of actinorhodin and undecylprodiginine assays of samples from S. coelicolor A2(3) M145 cultures grown in differently limited (N, P, C) Modified Evans Medium. [file 1756-0500-4-78-S5.PDF]
