# Supplementary material for: Genome-wide transcriptomic analysis of the response to nitrogen limitation in Streptomyces coelicolor A3(2)
Source: BMC Res Notes. 2011 Mar 23;4:78. doi: 10.1186/1756-0500-4-78 (PMC3073908; doi:10.1186/1756-0500-4-78)
Supplement: Additional File 7 — List of genes significantly differentially expressed at the N6/N7 time points relative to comparison time points. Details of their functional annotations and Expression Category (I, II or III) as defined in the "Results & Discussion" are provided. Those genes identified as a result of comparing the C-limited time points with the N-limited time points are marked *, those identified as a result of comparing the P-limited time points with the N-limited time points are marked # and those identified as a result of comparing the N1 nitrogen time point with subsequent N-limited time points are marked +. Unmarked genes were identified as a result of comparisons between the N- limited time points and both the C and P-limited time points. [file 1756-0500-4-78-S7.DOC]

**Additional File 7.**

| **Gene** | **Functional Annotation** | **Expression Category**  **(I, II or III)** | **Expression of gene at N6/N7**  **relative to corresponding P/C**  **time points** |
| --- | --- | --- | --- |
| SCO0097* | Integral membrane protein | I | Up |
| SCO0429* | Oxidoreductase with similarity to aldo/keto reductases | I | Up |
| SCO0682 | Hypothetical protein | II | Up |
| SCO0958^#^ | Hypothetical protein | I | Up |
| SCO1293 | Hypothetical protein with similarity to acetyltransferases & N-acetylglutamate synthetases | III | Up |
| SCO1366 | Hypothetical protein with similarity to transcriptional regulators | I | Up |
| SCO1397^#^ | Hypothetical protein with similarity to signal transduction proteins | I | Up |
| SCO1550 | Small membrane protein | I | Up |
| SCO1724 | Putative serine/threonine protein kinase | I | Up |
| SCO1860 | Putative secreted protein | III | Up |
| SCO1961^#^ | *aroQ*, dehydroquinate dehydratase | I | Up |
| SCO1998 | *rpsA*, 30S ribosomal protein S1 | I | Up |
| SCO2008 | Possible branched chain amino acid transporter | III | Up |
| SCO2471 | Possible secreted protein with similarity to proteins involved in cobalt metabolism | III | Up |
| SCO2486 | *nirB*, nitrate reductase | III | Up |
| SCO2487 | *nirB*, nitrate reductase large subunit | III | Up |
| SCO2862 | Hypothetical protein | I | Up |
| SCO2865* | Putative regulatory protein | I | Up |
| SCO2970* | Putative membrane protein | I | Up |
| SCO3113 | Transposase remnant | II | Up |
| SCO3215 | Hypothetical protein | I | Up |
| SCO3288 | Integral membrane protein | II | Up |
| SCO3289 | Large membrane protein | II | Up |
| SCO3323 | Putative RNA polymerase sigma factor | II | Up |
| SCO3327 | Small, hypothetical lysine/arginine rich protein | I | Up |
| SCO3328 | Hypothetical protein | I | Up |
| SCO3465* | Hypothetical protein with similarity to glycosyltransferases | I | Up |
| SCO3471 | *dagA*, extracellular agarase | I | Up |
| SCO3472* | Transposase remnant | I | Up |
| SCO3473* | Putative aldolase | I | Up |
| SCO3474 | Sugar kinase | I | Up |
| SCO3481 | Hypothetical protein with similarity to glycosyl hydrolases | I | Up |
| SCO3482 | Putative sugar permease | I | Up |
| SCO3487 | Possible hydrolase | I | Up |
| SCO3579 | Transcription regulatory protein | I | Up |
| SCO3662 | Hypothetical protein | I | Up |
| SCO3663 | Possible membrane protein | I | Up |
| SCO4187 | Putative membrane protein | I | Up |
| SCO4295 | *scoF4*, cold shock protein | I | Up |
| SCO4425^#^ | *afsR2*, sigma factor like protein | II | Up |
| SCO4505* | *scoF2*, cold shock protein | I | Up |
| SCO4677 | Anti-sigma factor regulatory protein | II | Up |
| SCO4725 | *infA*, translational initiation factor | I | Up |
| SCO5083^#^ | *actII*, putative actinorhodin transporter | II | Up |
| SCO5145 | Hypothetical protein | I | Up |
| SCO5163 | Hypothetical protein | III | Up |
| SCO5321* | Polyketide hydroxylase | I | Up |
| SCO5583 | *amtB* ammonium transporter | III | Up |
| SCO5584 | *glnK*, PII signal protein | III | Up |
| SCO5585* | *glnD*, adenylyltransferase | I | Up |
| SCO5592^#^ | Hypothetical protein with similarity to RNA binding proteins | I | Up |
| SCO5705 | Hypothetical protein | I | Up |
| SCO6282^#^ | 3-oxoacyl-[acyl carrier protein] reductase | II | Up |
| SCO6624* | Putative membrane protein | I | Up |
| SCO6682 | Hypothetical protein | I | Up |
| SCO6794^#^ | Putative membrane protein | I | Up |
| SCO6796 | Hypothetical protein | III | Up |
| SCO6797* | Possible ATP/GTP binding protein | I | Up |
| SCO6875* | Hypothetical protein | I | Up |
| SCO6931 | Hypothetical protein | II | Up |
| SCO7251^#^ | Hypothetical protein | I | Up |
| SCO7636* | Possible membrane protein | I | Up |
|  |  |  |  |
| SCO1629^+^ | Possible oxidoreductase with similarity to short chain dehydrogenases | II | Up |
| SCO3290^+^ | Hypothetical protein | II | Up |
| SCO5254^+^ | *sod*, superoxide dismutase | II | Up |
| SCO5582^+^ | Possible regulator with similarity to sporulation associated protein | II | Up |
| SCO5443^+^ | *pep1A*, possible alpha-amylase | II | Up |
